# A Collagen–Elastin Regenerative Dermal Matrix May Generate Unfavorable Results in Head and Neck Postburn Scar Reconstruction: A Case Series

**DOI:** 10.3390/medicina61040744

**Published:** 2025-04-17

**Authors:** Bogdan Nitescu, Andrei Dumitrescu, Florin Radu Stanescu, Diana Cintacioiu, Gratiana Lates, Sorin Viorel Parasca

**Affiliations:** 1 Faculty of Medicine, University of Medicine and Pharmacy “Carol Davila”, 050474 Bucharest, Romania; Andrei.dumitrescu@drd.umfcd.ro (A.D.); florin-radu.stanescu@rez.umfcd.ro (F.R.S.); diana-georgiana.cintacioiu@rez.umfcd.ro (D.C.); sorin.parasca@umfcd.ro (S.V.P.); 2 Clinical Emergency Hospital of Plastic, Reconstructive Sugery and Burns, 050474 Bucharest, Romania; grati.lates@gmail.com

**Keywords:** collagen–elastin dermal matrix, burn scars, complications, reconstructive surgery, severe burns, face and neck burns, unfavorable results, head and neck

## Abstract

*Background and Objectives:* Dermal matrices have brought solutions for many problems, mainly in the treatment of burns and burn scar revisions. The objective of this study was to draw attention to the limits of a collagen–elastin dermal matrix (MatriDerm^®^) in its 1 mm variant for the treatment of burn scars on the face and neck. *Materials and Methods:* A case series of four patients (three women and one man) with burn scars of the face (one case) and of the neck (three cases) treated with collagen–elastin matrices is presented. In all cases, the excision or release of the scars was performed, and the defects were covered with MatriDerm^®^ and thin split-thickness skin grafts in the same operative time. *Results:* In all cases, the graft take was very good but was followed by the important contraction of the graft to such an extent that the results were found to be poor by both the surgeons and the patients. The surface of the new scar was irregular, and the elasticity was low. The article points out some probable causes and draws attention to the need for more objective studies regarding the use of this dermal matrix in burn scars of the head and neck. *Conclusions:* This collagen–elastin 1 mm dermal matrix should be used with caution for the surgical treatment of burn scars of the head and neck area, and its indication should be carefully weighted.

## 1. Introduction

Burns are one of the most challenging forms of trauma, but important technological progress has been made in the last half-century that has led to both improved survival rates and quality of life for survivors. Among the therapeutic armamentarium, dermal substitutes (acellular dermal matrices) have gained an important place. Their advantage is that they can be integrated by the body, producing a neo-dermis that can be grafted with very thin split-thickness skin grafts (STSGs), allowing for the repeated harvesting of grafts from the same donor area and producing better postburn scars.

One of the most popular synthetic dermal matrices is MatriDerm^®^ (MedSkin Solutions, Dr. Suwelack AG, Billerbeck, Germany). It is a single-layer dermal substitute composed of two elements: bovine collagen (types I, III, V) and hydrolysate elastin. Although it obtained its FDA (Food and Drug Administration) approval in 2021, on other continents (especially Europe), MatriDerm^®^ has been used in both clinical studies and common practice for acute burn grafting with great reported success [1]. One of the main advantages that MatriDerm^®^ has among other dermal matrices is that it has a variant (1 mm thick) which can be grafted immediately with skin grafts in the same operation. This is probably due to the fact that the collagen in this product is not cross-linked, which allows for quick absorption [2]. In acute burns, this allows for the rapid closure of the excised wound, making the grafted area more resistant to infections. In a meta-analysis published by van den Bosch et al. [3], MatriDerm^®^ with STSGs needed longer healing times than STSGs alone but produced better scars. Among the treated areas, the upper extremities seem to show the best results, but the head and neck areas also benefited from this method, with good results even in children [4,5,6].

There are fewer reports regarding MatriDerm^®^ use in postburn scars (seven, as identified in [3]). In this meta-analysis, the upper extremities were the most frequent site for reconstruction with a collagen–elastin dermal matrix (CEDM). Correa et al. [7] reported more contraction in burn scars treated with MatriDerm^®^ than ones treated with STSGs or Integra^®^ in a controlled prospective study. In a similar study, which included histological evaluation, Almeida et al. [8] did not find any advantage of dermal matrices over STSGs in burn scars. Postburn scars of the head and neck are probably the most challenging due to both esthetic and functional reasons. Classical ways of reconstruction are based on full-thickness skin grafts for the face and split-thickness skin grafts for the neck, but the latter tend to contract and are prone to hyperpigmentation [9]. Local thin flaps, pre-expanded or not, are reported to provide good results for neck contractures, but are seldom available [10]. Free flaps are also used, but the different skin texture, the risk of losing the whole flap and the length of the surgery are serious disadvantages [10]. That is why dermal matrices seem like a good solution for the head and neck; yet, very few reports have been published on this topic. Seo et al. [11] reported excellent and good results in 28 patients with matured neck contractures treated with dermal matrices (13 benefited from CEDM and 15 from AlloDerm^®^) and STSGs. Yamur et al. also described favorable outcomes in eight pediatric patients with scars of the head and neck [12].

The aim of this article is to present a mini case series in which MatriDerm^®^ was used to treat burn scars of the head and neck with poor results, and to draw attention to the need for more thorough research regarding the indications of the product in this area.

## 2. Materials and Methods

A retrospective observational study of the patients treated for burn scars of the head and neck regions using collagen elastin dermal matrices at the Teaching Emergency Hospital for Plastic Surgery and Burns, Bucharest, Romania, between 2018 and 2023 was conducted. The charts of the patients, alongside available photographic material, were reviewed, and several pieces of information were gathered: age and sex, the time spent healing until MatriDerm^®^ was used, a description of the scars, operative notes, the evolution of the treated scars. The follow-up period was between 6 and 9 months. A short case series emerged from the study.

The case series consisted of 4 patients (3 women and 1 man) who were treated in the Teaching Emergency Hospital for Plastic Surgery and Burns, Bucharest, Romania. One (25 years old) had old postburn scars on the face and hands and was treated for a left cheek scar and upper lip ectropion (see case 1), while the other three were 38, 42 and 54 years old and were treated for anterior neck contracture (2 patients) and right lateral neck contracture. Patients were admitted to our burn ward and were treated by our surgical team.

All the patients gave their written informed consent for the procedure as well as for the use of their photos for scientific purposes, including publication. All the procedures respected the patients’ rights according to the Helsinki Declaration and subsequent regulations and were approved by the hospital’s Ethics Committee (resolutions no 374/2018, date: 11 July 2018; no 174/2022, date: 11 April 2022).

## 3. Results

The patients were treated with the complete excision of the scar (three patients) or releasing incisions, and the defects were covered with 1 mm MatriDerm^®^ and split-thickness skin grafts (0.15 mm thick) from the thighs (avoiding old donor areas). In all patients we observed good graft take at 10 days (more than 90% of the grafted area). Three patients benefited from compressive therapy and silicone gel applications in the following months. In all patients, significant graft contraction was noted at 9–12 months, with little relief of symptoms or improvement in scar appearance.

### 3.1. Case 1

**Figure 1:**
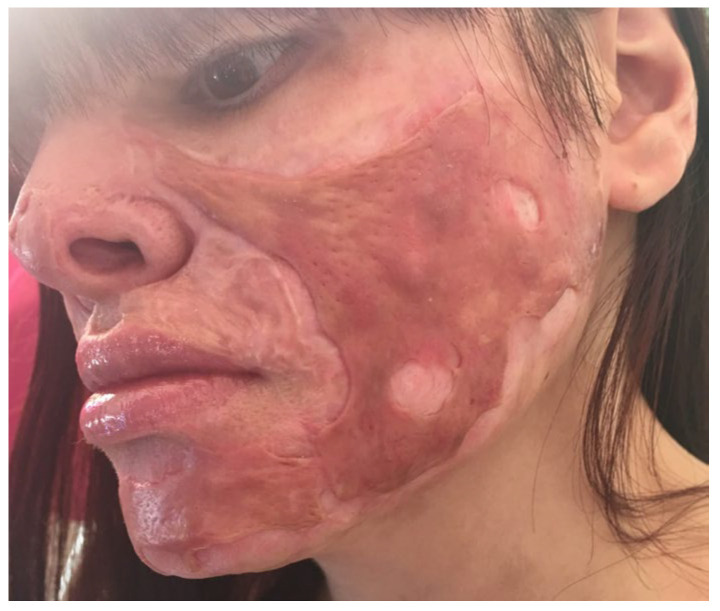
The preoperative view of the patient from case 1: hypertrophic scars after dermabrasion and overgrafting an old burn scar on the cheek and ectropion of the upper lip.

A 25-year-old female patient with burn scars on the head (mostly on the face) and both hands after a burn at the age of three, was admitted to the hospital. After the patient repeatedly refused an expander on the neck (which was free of scars), we decided to address the left cheek scar and the upper lip ectropion with excision and grafting using 1 mm Matriderm^®^ and a thin STSG from the thigh. The scar on the left cheek (after a previous unsuccessful attempt of resurfacing with dermabrasion and overgrafting 10 months before) occupied almost all the esthetic unit of the cheek and preauricular area (with a narrow strip of normal skin along the mandible line) and showed large areas of hypertrophy, while the upper lip scar was contracted, irregular and exposed the upper lip vermilion in excess (Figure 1).

**Figure 2:**
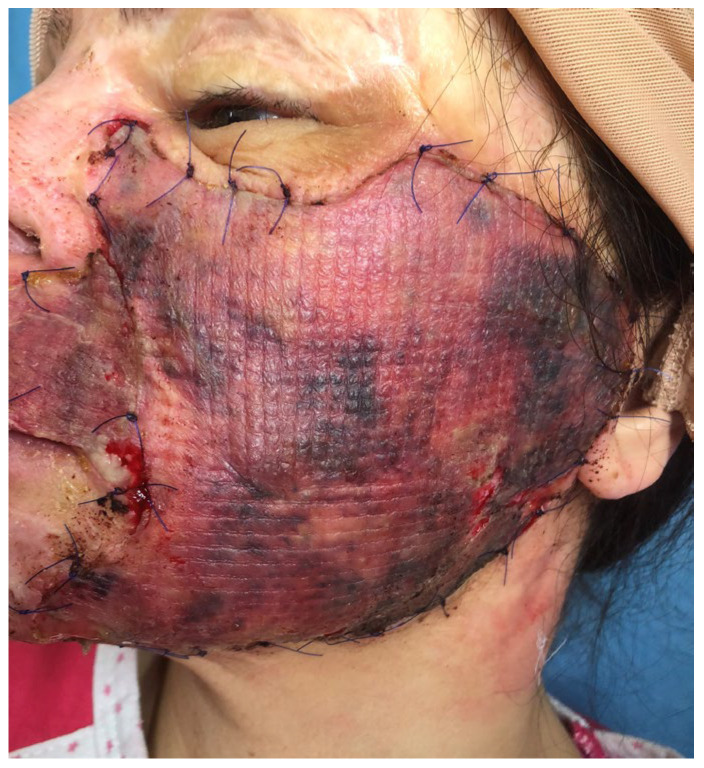
Patient from Figure 1 on fifth post-operative day: good integration of graft with some areas of graft cyanosis.

**Figure 3:**
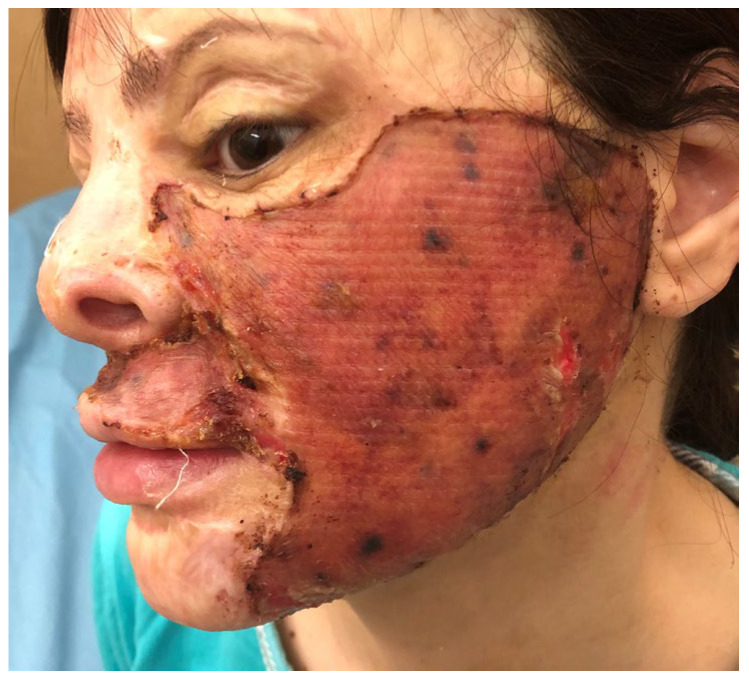
Patient 1 on 10th post-operative day: good graft take in whole treated area.

The surgery was performed under general anesthesia. The scars from the left cheek and the upper lip were completely excised, respecting the esthetic subunits, and any contracting bands were released. After hemostasis, a 1 mm CEDM was applied and hydrated after application with normal saline. The 0.15 mm thick STSG was applied over the Matriderm^®^ and fixed with separated sutures. The grafted area was larger than the initial scar, extending inferior to the mandible line with no tension. The graft was dressed with tulle gras and wet tissue and a slight compressive dressing was used on the outer side. The patient was put on a soft-food diet and was instructed to avoid mandible and upper lip movements. On day 5, the dressing was changed and showed a good take of the skin graft, with some areas of cyanotic skin, mainly in the preauricular area (Figure 2). At 10 days post-operation, the graft was completely revascularized on the whole treated area (Figure 3). The patient was discharged and instructed to wear a compressive elastic mask and apply silicone gel daily.

**Figure 4:**
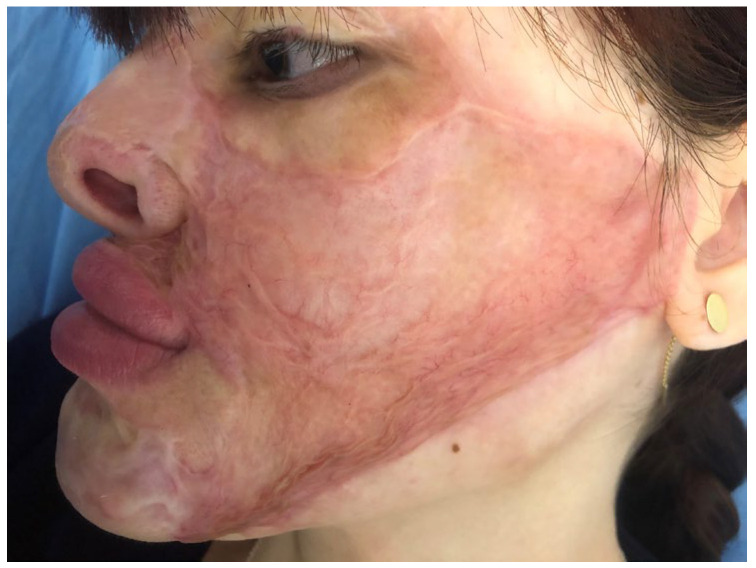
Patient 1 at 3 months: the important contraction of the graft both on the cheek and upper lip with hypertrophy along the mandible.

**Figure 5:**
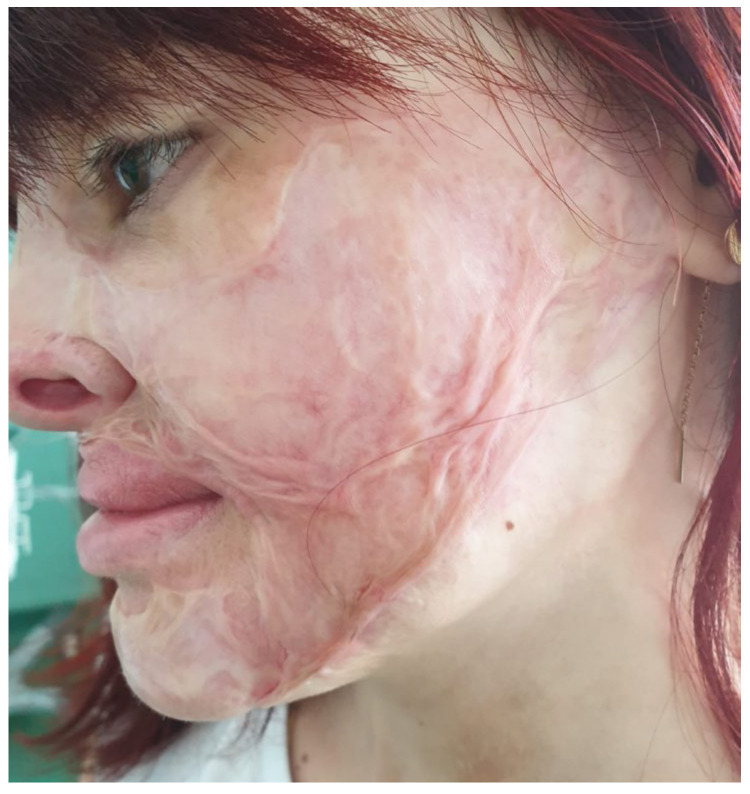
Patient 1 at 1 year: the unsightly appearance of the scar on the cheek with contraction, hypertrophy and an irregular surface; upper lip ectropion looks worse than preoperatively.

At three months, the new scar displayed significant contraction along the mandible line, with hypertrophy at the edge and huge contraction on the upper lip, which seemed to look even worse than it did pre-operatively (Figure 4). Triamcinolone injections were performed in the hypertrophic band and the patient was instructed to continue with compressive therapy and silicone gel. At twelve months, the hypertrophic bands were still visible, starting in the preauricular area and bifurcating anteriorly towards the left buccal commissure and the mental area. The rest of the scar on the cheek regained normal color, but the texture was considered poor both by the patient and the surgeon (Figure 5). The upper lip was slightly improved compared with 3 months post-op. Although the scars looked better than before surgery, the hypertrophy and significant contraction along the mandible line and the uneven surface of the rest of the cheek made us consider the result to be poor. No improvements were noted at following visits, despite two more injections with triamcinolone.

### 3.2. Case 2

**Figure 6:**
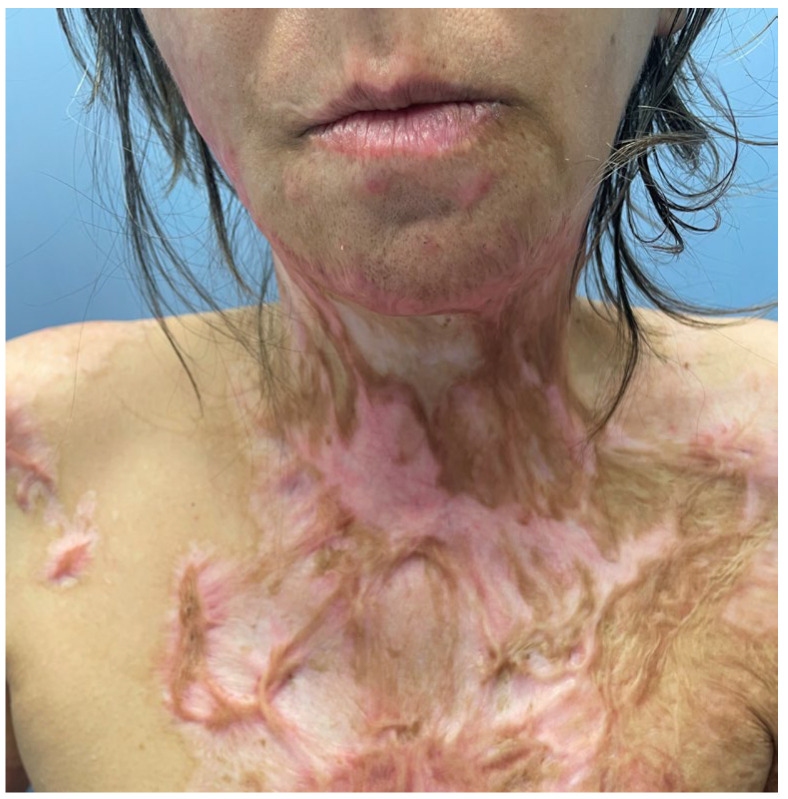
Patient 2 before surgery: retraction of anterior neck scar with areas of hypertrophy.

A 42 year-old female patient, 7 months after a self-inflicted 60% total body surface area burn treated in another facility, was referred to our hospital for contracture of the anterior neck, with severe limitation of neck movement and areas of hypertrophy that were itchy and sometimes painful (Figure 6). At that time, the patient was serving a conviction in a state penitentiary. Because the scar was not mature, we decided to perform a limited release of the scar, with which we hoped to reduce tension and reduce the hypertrophy. We were aware that the patient’s judicial situation would be deleterious to their late post-operative care. We decided to use Matriderm^®^ for the defect resulting from the release in the hope that the new dermis would help in reducing the post-operative contraction of the wound.

**Figure 7:**
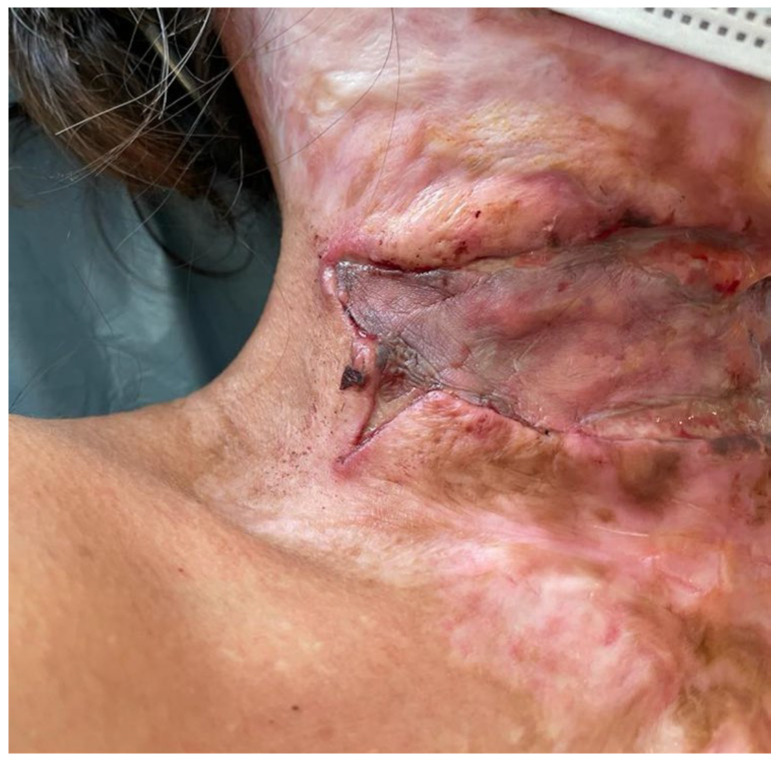
Patient 2 at 5 days post-op. Right side of graft well integrated.

**Figure 8:**
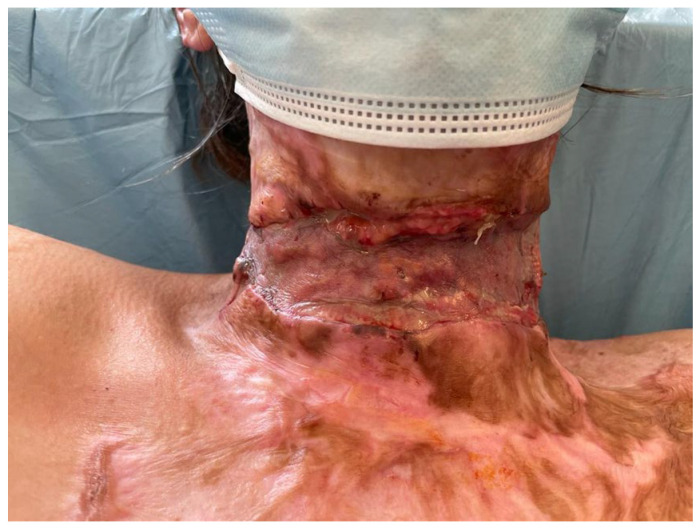
Patient 2 at 10 days: good graft take, with some graft lost at upper part of defect.

The procedure was performed under general anesthesia. A horizontal incision was performed on the neck scar at the level of the thyroid cartilage. No platysma muscle was found, and the incision was carried out into healthy subcutaneous tissue. On the lateral parts, the incision reached healthy skin, where it was bifurcated for 1–2 cm. The neck was straightened as much as possible and a 5 cm per 18 cm defect resulted. Hemostasis was performed with a cautery. The resulting wound was covered with 1 mm Matriderm^®^ and a 0.15 mm STSG from the thigh. The graft was sutured into place and dressed with a bulky dressing and a collar. At 5 days, the graft take was good (Figure 7). At 10 days, the graft was integrated, with the exception of a narrow strip (approximately 1 cm wide) at the upper part of the defect (Figure 8). Although the patient was instructed to wear a collar and use silicone gel, that was not always possible in the state penitentiary.

**Figure 9:**
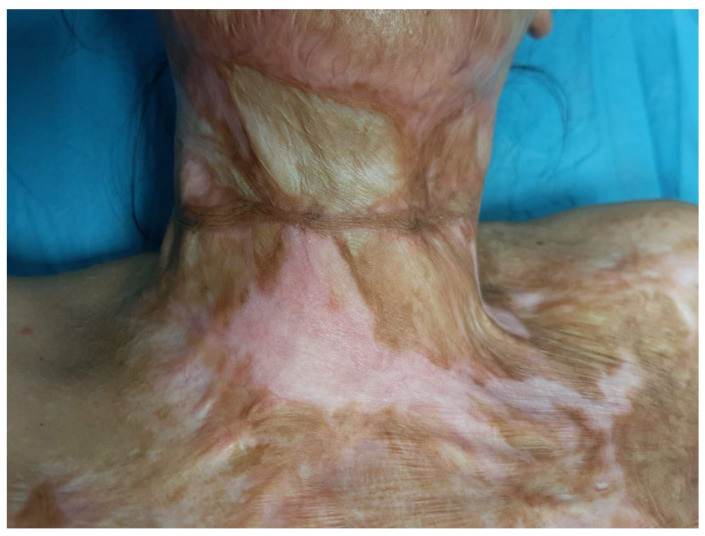
Patient 2 at 6 months: major contraction of graft to a width of less than 1 cm; fewer signs of hypertrophy of postburn scar.

At the 6-month visit, the graft had significantly contracted to a width of less than 1 cm and was hard and hyperpigmented. The postburn scar of the neck was softer, with less hypertrophy, and the patient reported slight improvement in neck movement (Figure 9). With such a contraction of the Matriderm^®^, the result was considered poor by surgeons. After her release from the prison, the patient received further treatment for the scar, with full excision and a thick STSG, that provided a good result.

### 3.3. Case 3

**Figure 10:**
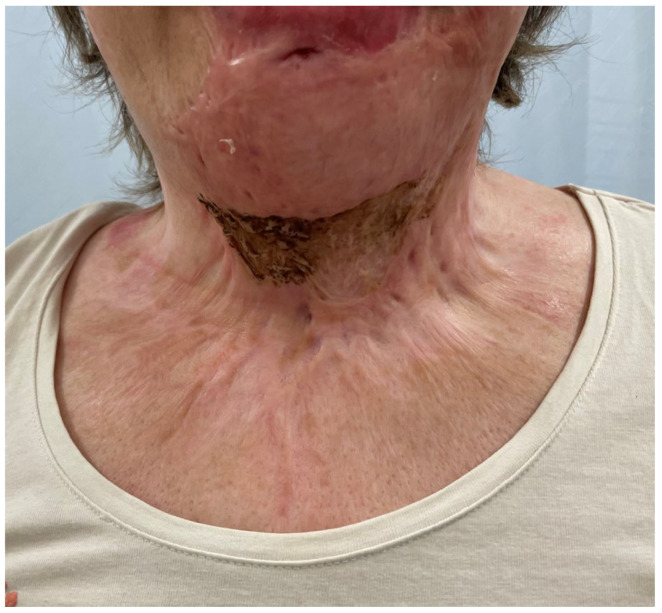
The preoperative view of the anterior neck contracture of patient 3; note the hypertrophy of the scar and the unfavorable aspect of the graft in the center.

A 38-year-old woman treated for flame burns on 40% of the body in another facility, was admitted to the hospital 6 months after the burns were completely healed. After three months of healing, she had a release of an anterior neck contracture with an STSG. The contracture relapsed, and despite the fact that the scars were not matured, we decided to operate in order to improve her quality of life. At clinical examination, a hypertrophic scar covered the lower lip, menton and upper part of the anterior neck. Inferior to this, a contracted, hyperpigmented, irregular postgrafting scar was seen. In the inferior part of the neck, caudal to the graft scar, another irregular and hypertrophic postburn placard could be seen. The patient had impaired movement of the neck with severely limited extension (Figure 10).

**Figure 11:**
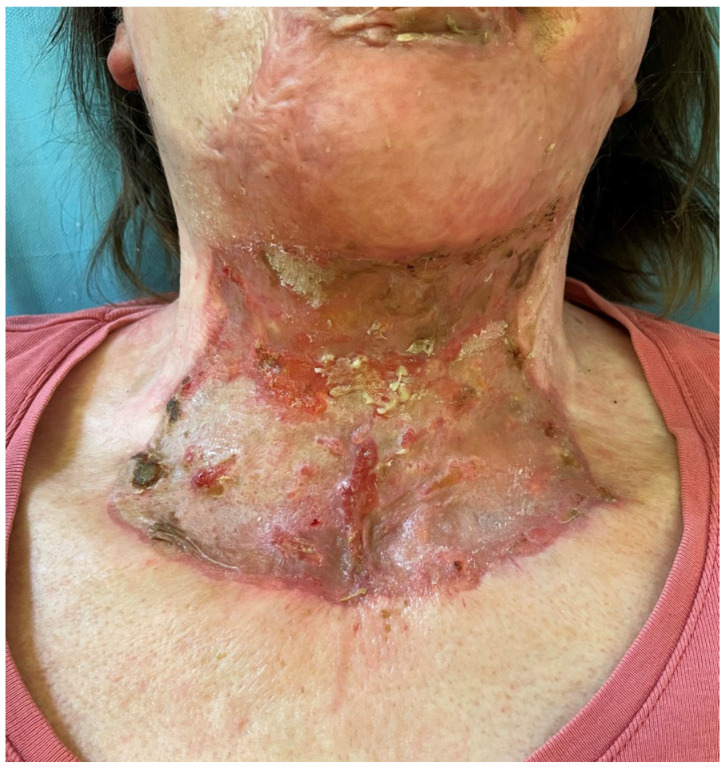
The postoperative view of the graft (patient 3) at 12 days: good graft take with a few scattered small granulating wounds that were left to heal spontaneously.

The whole scar covering the anterior part of the neck was excised into healthy tissue and the contracture was released completely. The defect (approximately trapezoidal, with an upper base about 10 cm, lateral lines about 12 cm and an inferior base of 15 cm) was covered with 1 mm MatriDerm^®^ and a 0.15 mm STSG harvested from an unscarred area of the thigh. A conformateur was applied. The dressings were changed at 5 days and then every two days until healing. At 12 days (Figure 11), the graft showed good take (about 80%), with islands of granulating tissue. After full healing, the patient wore a silicone neck conformateur and silicone gels were used on a daily base.

**Figure 12:**
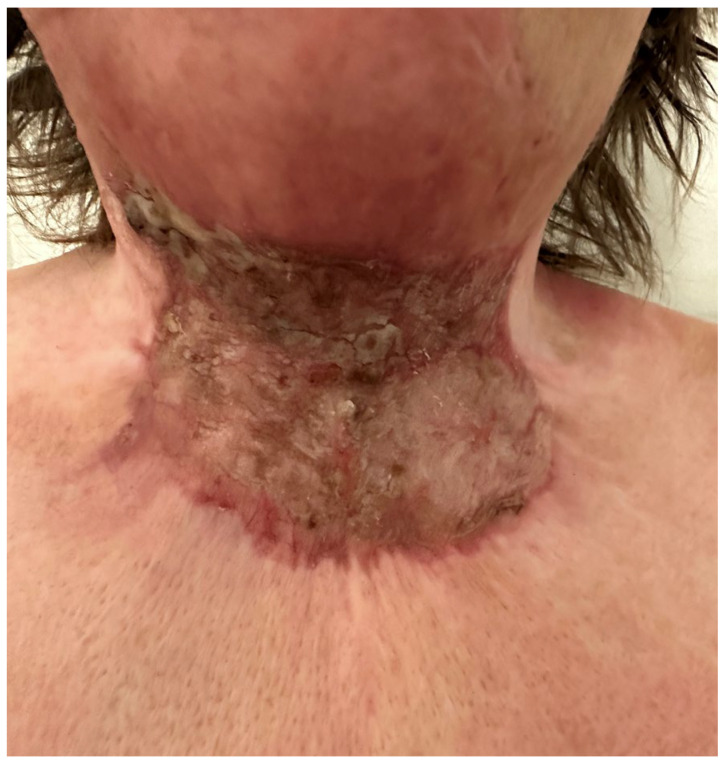
A postoperative photo of patient 3 at 3 months, showing important graft contraction, hyperpigmentation and poor graft texture.

At the 3-month follow-up, the graft had contracted to almost one half of its original size and had a hyperpigmented appearance with hyperkeratotic areas (Figure 12). The movement limitation was almost the same as before surgery. The patient was instructed to continue with conservative anti-scar therapy and to double the applications of silicone gels.

**Figure 13:**
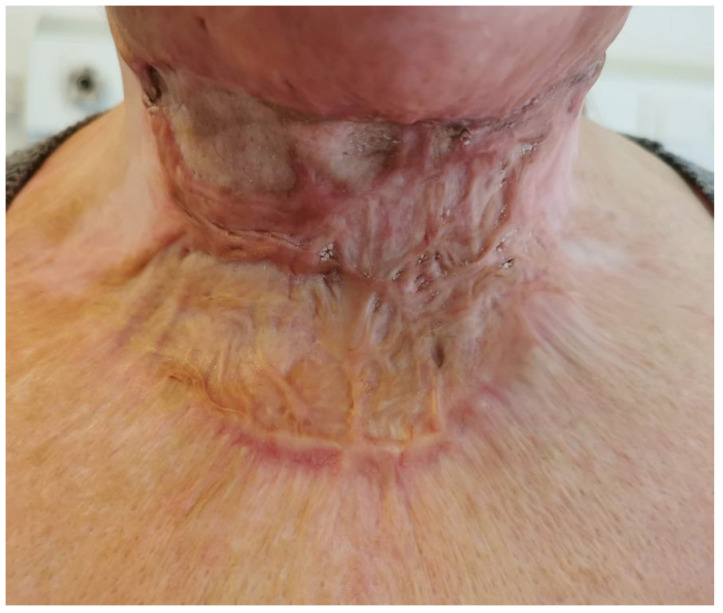
The postoperative view of patient 3 at 9 months, showing slight improvement in contracture compared to Figure 12 but a poor esthetic outcome, with an irregular surface and signs of hypertrophy.

At 9 months, the scar showed some improvement regarding contraction but had a wide area of hypertrophy. The movement of the neck was still seriously impaired (Figure 13). Both the surgeons and the patient were disappointed by the results, and the patient was further treated with the excision of the scar, covering it with a supraclavicular flap from the right shoulder.

### 3.4. Case 4

**Figure 14:**
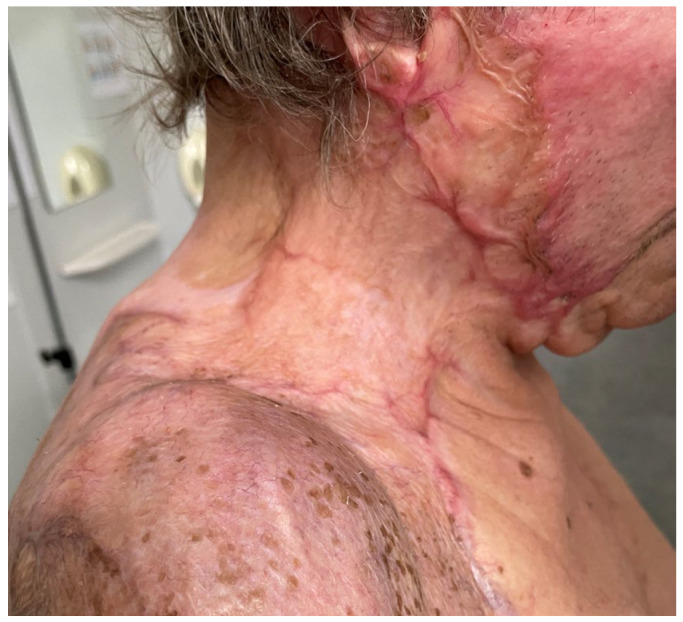
A preoperative view of patient 4, with a right lateral neck contracture and slight hypertrophy.

A 54-year-old male was admitted to our department for scars after a 25% TBSA burn healed 9 months prior. The patient complained about a right lateral contracture of the neck with movement limitation. The scar was 7 over 10 cm with few signs of hypertrophy, mainly on the anterior border (Figure 14).

The scar was completely excised, and the defect was closed with the same combination of 1 mm MatriDerm^®^ and a thin STSG. Two releasing incisions, perpendicular to the anterior side of the defect, were performed and grafted in the same way. The same post-operative protocol was used as in the previously presented cases. Anti-scar conservative therapy was employed.

**Figure 15:**
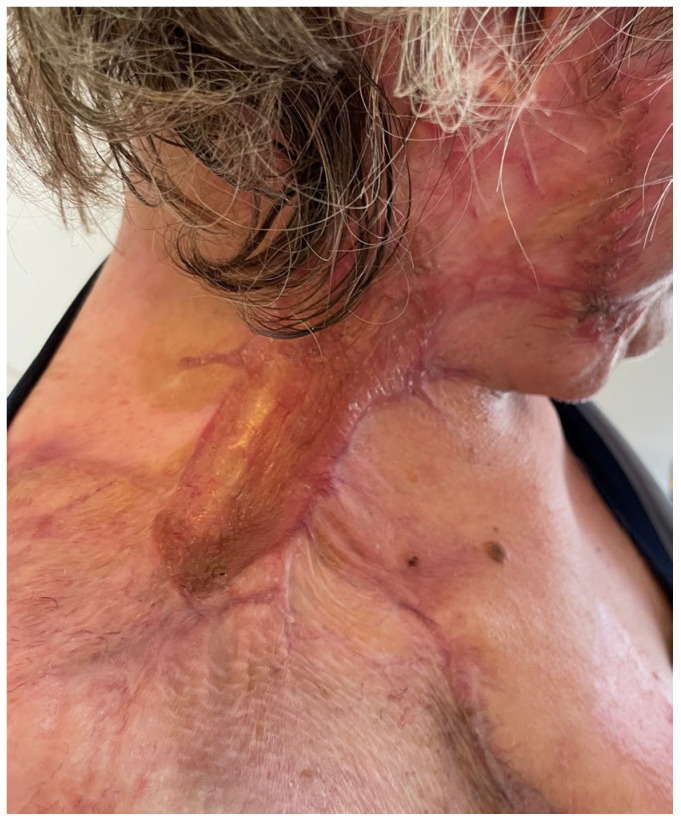
A postoperative view of patient 4 at 4 months, showing the significant contraction of the Matriderm^®^ skin graft and hypertrophy.

At the 4-month follow-up, the resulting scar was significantly contracted (to about 50% of the original defect), with important hypertrophy and complete contraction of the grafts from the releasing incisions at the anterior side of the scar. No improvement of neck movement was detected (Figure 15).

**Figure 16:**
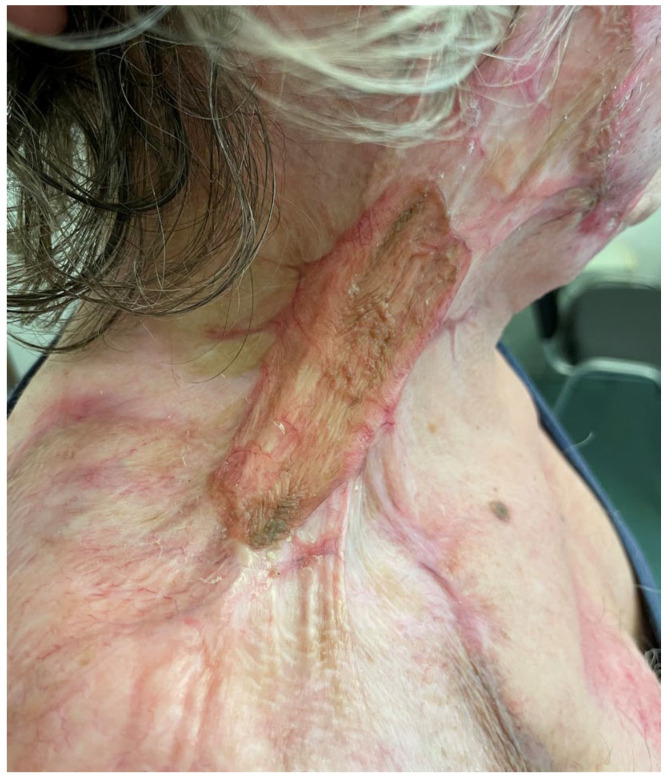
A postoperative view of patient 4 at 9 months, with diminished hypertrophy but significant contraction and a poor esthetic result.

At 9 months, the scar was still contracted; the level of hypertrophy had diminished, but the surface was irregular and unsightly (Figure 16). Still no improvement in neck movement was noticed. The result was considered poor both esthetically and functionally. The patient was further treated with a combination of Z-plasties and STSGs.

Important contraction of the 1 mm MatriDerm^®^ with STSGs was noted in all cases. The surfaces of the resulting scars were irregular and there were no noticeable functional or esthetic improvements. Therefore, we decide to abandon this line of treatment for similar cases.

## 4. Discussion

The article describes a mini case series of postburn scars of the face or neck that were treated with collagen–elastin dermal matrices and STSGs and produced unfavorable results. In the case of the face scar, despite good graft take and an initial favorable evolution, significant contraction of the new scar resulted at one year, with an unsightly appearance and upper lip ectropion worsening. While the scar’s contraction was beneficial for the patient as it expanded the normal skin from the neck to the treated area (cheek), the final scar appearance was poor. On the cheek, the surface texture improved, but the important contraction of the scar, mainly on the upper lip, meant that both the patient and the surgeons were unhappy with the result. It is difficult to explain such a result in a matured scar which was completely excised and benefited from pressure therapy and silicone gel applications. Hypertrophy of the scar was detected along the mandible line and required treatment with triamcinolone injections. Disappointed by this result, we have not used this type of dermal matrix in facial burn scars reconstruction since then. There is only one report of the use of this dermal matrix for burn scars of the face in children [12], and none in adults, although good results were reported about its use in acute facial burns [5,6].

The other three cases described the use of Matriderm^®^ in postburn neck contractures with poor results and little contracture relief. The unsuccessful outcomes may have some explanations in these cases: the scars were not matured, the treatment consisted only of contracture release without full scar excision in case 2 and, in the same case, no conformateur or other conservative anti-scar contracture measure was used. In fact, the aim of the surgery was to release the tension of the scars and diminish their contraction while they were still in the period of evolution. We also hoped that the neo-dermis would produce a slight improvement in movement, which could have helped the patients to pass through this period more easily. The contraction of the new graft was so spectacular that it seemed to contradict the expectations of a product that turns a thin STSG into one that is thick and less prone to contraction. A similar case was presented by Greenwood and Mackie [13], but they performed a full scar resection with platysmectomy and post-operative negative-pressure therapy with significant graft contracture and reported improvements in appearance, scar elasticity and neck movements. The authors were surprised by the difference in scar dimensions between the early post-operative stage and 4 months after the operation, but declared themselves satisfied by the result. Seo et al. also reported on a case series of postburn neck contractures treated with dermal substitutes including MatriDerm^®^ [11]. They found their results to be excellent or good, while the complication rate was rather low (one infection in 28 patients). In Seo’s study, the scars were operated on after 1 year and the full excision of the scars was performed before grafting. Negative-pressure wound therapy was also applied in the post-operative period. We did not use negative-pressure therapy for the grafts, but the graft take was very good in our cases, too, so the poor results we had do not seem to have been caused by our early post-operative approach. Good results were reported for face and neck scars in eight children by Yagmur and colleagues [12]. The scars improved in texture and elasticity. Some contraction of the grafts was also reported (less than 10%), but no neck contractures were treated.

In a more recent comparative study by Almeida et al. [8], various dermal matrices (including CEDM) were found to have no advantage when compared to single STSGs for the treatment of burn scars in various locations. A previous report by Correa et al. [7] demonstrated the important contraction of MatriDerm^®^ with STSGs compared with STSGs alone or Integra^®^ and STSGs, results that are compatible with our own findings. They report an average of 59.17% graft contraction for the MatriDerm^®^ group for various locations of scars. In a more recent study, Vana et al. [14] compared 2 mm MatriDerm^®^ and Integra^®^ in treating burn contractures in various parts of the body, including three neck cases. Both matrices were grafted at 3 weeks. They report more retraction in the MatriDerm^®^ cases and poorer skin quality according to the Vancouver and POSAS scales. Yet, both matrices provided improvement in the scars.

Although our report is far from being a proof, collagen–elastin acellular dermal matrix should probably be used with caution in neck contractures and only after full scar excision followed by aggressive scar management. And even such an approach could generate sub-optimal results in the face. One cannot rely only on the products’ ability to stimulate dermal regeneration for preventing scar contracture, at least in similar clinical cases. In the histological study by Dickson et al. [2], early resorption of the dermal matrix and replacement by the neo-dermis is reported, with more inflammation compared to Integra^®^, a fact that might partially explain the significant scar contraction. They also reported no improvement in burn scars at 12 months using mVSS scores.

The major limitation of our report is the small number of patients. We actually gave up this line of treatment after consecutive disappointing cases; therefore, we could not report more cases. The report is not comparative, so any conclusions are based more on expectations. The clinical course was, though, consistently similar in all cases, and the contraction of the grafts was significant. We also did not perform any quantitative evaluation of the pre- and post-operative scars, but both the surgeons and the patients agreed that the results were sub-optimal. Our main intention was to draw attention to the fact that some applications of MatriDerm^®^ in certain areas with complex movements (like the head and neck) might produce unexpected poor results, and to stimulate more comparative prospective studies which can bring more reliable information about its indications in different areas of the body.

The article would have benefited from a review of the literature about MatriDerm^®^ applications for burn scars, but there have been so many reviews on the products’ uses published recently, yet so few reports about its value for treating burn scars, and the most relevant articles were mentioned in this section.

## 5. Conclusions

Our study reports four consecutive cases of burn scars of the face and neck in which a 1 mm collagen elastin dermal matrix and an STSG generated important retraction and unsightly scars with minimal improvement. This points out the possible limitations of this dermal matrix regarding these particular locations. Reporting unfavorable results can bring valuable knowledge about this method and its indications, even though more comparative studies are needed in order to generate indisputable conclusions regarding this subject.

## Data Availability

The data presented in this study are available on request from the corresponding author.

## Abbreviations

The following abbreviations are used in this manuscript:

MDPI	Multidisciplinary Digital Publishing Institute
STSG	split-thickness skin graft
FDA	Food and Drug Administration
CEDM	collagen–elastin dermal matrix
